# Isolation and identification of *Komagataeibacter xylinus* from Iranian traditional vinegars and molecular analyses

**Published:** 2017-12

**Authors:** Paria Sadat Lavasani, Elahe Motevaseli, Mahdieh Shirzad, Mohammad Hossein Modarressi

**Affiliations:** 1 Department of Molecular Medicine, School of Advanced Technologies in Medicine, Tehran University of Medical Sciences, Tehran, Iran; 2 Department of Microbiology, School of Biology, College of Sciences, Tehran University, Tehran, Iran; 3 Department of Medical Genetics, School of Medicine, Tehran University of Medical Sciences, Tehran, Iran

**Keywords:** *Komagataeibacter xylinus*, *16S rRNA*, *bcsA*, *pheS*, *rpoA*

## Abstract

**Background and Objectives:**

Acetic acid bacteria (AAB) are one of the major interests of researchers. Traditional vinegars are suitable sources of AAB because they are not undergone industrial process like filtering and adding preservatives. *Komagataeibacter xylinus* as a member of AAB is known as the main cellulose producer among other bacteria. The purpose of the current study was to isolate the bacteria from traditional vinegars and its molecular analyses.

**Materials and Methods:**

Vinegar samples were collected. Well-organized bacteriological tests were carried out to differentiate isolated bacteria from other cellulose producers and to identify *K. xylinus*. NaOH treatment and Calcofluor white staining were used for detecting cellulose. Chromosomal DNA of each strain was extracted via three methods of boiling, phenol-chloroform and sonication. Molecular analyses were performed on the basis of 16S rRNA sequences and cellulose synthase catalytic subunit gene *(bcsA)* for further confirmation. Phylogenetic tree was constructed for more characterization. Two housekeeping genes were studied including phenylalanyl-tRNA synthase alpha subunit *(pheS)* and RNA polymerase alpha subunit *(rpoA)*.

**Results:**

Of the 97 samples, 43 *K. xylinus* strains were isolated. They were identified via bacteriological and molecular techniques. 16S rDNA sequence showed 99% similarity with registered sequences of the bacteria. Biodiversity of the genome confirmed by analyzing *bcsA, pheS* and *rpoA* genes.

**Conclusion:**

*K. xylinus* can be isolated from traditional vinegars. Screening tests ought to include the classical methods and molecular techniques. Different molecular techniques and more genomic research should be developed to expand our knowledge for distinguishing isolated bacteria especially in the fields of AAB.

## INTRODUCTION

Acetic acid bacteria (AAB) are widely used in food and beverage production industry. Vinegars, one of those beverages, are produced in traditional and industrial forms. Traditional vinegars have wide range of different types of microorganisms in comparison to those undergone industrial process. Isolating new strains from traditional vinegars is a subject of interest because of their high resistance to stress such as acidity and temperature during fermentation. One of the bacteria can be isolated from traditional vinegars is *K. xylinus* (1–3).

*K. xylinus* (previously named *Gluconacetobacter xylinus*) is a rod-shaped aerobic microorganism, Gram negative and catalase positive. The bacterium is normally found in vinegars, fruits and vegetables. It is able to secrete a thick gelatinous coating layer on the surface of the liquid medium which is considered cellulose (4–6).

Cellulose is the main homo-biopolymer on the Earth composed of repeating unit of B-D gluco-pyranose (7). It is presented in a wide range of living organisms from plants to bacteria (8). The unique structure of cellulose makes it suitable as a multi-purpose material with potential application in allied sciences such as food production, medical applications and paper industries (9, 10).

Bacterial cellulose with the for mulation of (C_6_H_10_O_5_)n is an extracellular polysaccharide produced by different species of bacteria such as *Rhizobium leguminosarum, Agrobacterium tumefaciens*, *Escherichia coli* and *Salmonella enterica. K. xylinus* belonged to acetic acid-producing genera are known as a model microorganism for producing bacterial cellulose. Cellulose obtained from the bacteria is free from lignin, hemi-cellulose and pectin. This pure structure makes the network ultra-fine and unique for industrial applications (10–12).

Biochemical and physiological tests play important roles in identification and differentiation of new isolates especially when they are taken from natural sources like traditional vinegars. Final confirmation of isolates should be obtained by molecular analyses using polymerase chain reaction amplification and 16S rRNA sequencing (13).

This research was performed in order to isolate some strains of *K. xulinus* from Iranian traditional vinegar samples. Biochemical and physiological tests were carried out for differentiation of the bacteria from other bacteria known as cellulose producers. Isolates were characterized based on 16S rRNA sequence. Cellulose production of the isolated bacteria was investigated using Calcofluor white staining and NaOH treatment. In addition, molecular analyses were performed based on cellulose synthase catalytic subunit and two housekeeping genes including phenylalanyl-tRNA synthase alpha subunit *(pheS)* and RNA polymerase alpha subunit *(rpoA)*.

## MATERIALS AND METHODS

### Sample collection and culture condition.

97 samples of traditional vinegars were collected from different provinces of Iran from January to June 2014. Isolated strains were obtained according to the protocol described below. Briefly, 1 ml of each sample was individually cultured on solid Hestrin-Schramm (HS) medium containing D-glucose 2.0%, peptone 0.5%, yeast extract 0.5%, Na_2_HPO_4_ 0.27%, citric acid 1.15% and agar 1.5%. All chemicals were purchased from Sigma Aldrich. The plates were incubated at 28–30°C for 48 h (13–15). White to cream grown colonies with mucosal structure were considered *K. xylinus* which were able to produce cellulose and were stored in skim milk medium for further analyses. Reference strain (*Gluconacetobacter xylinus*; PTCC No.: 1734) was purchased from Iranian Research Organization for Science and Technology (IROST).

### Biochemical and physiological tests.

The isolated bacteria were stained with conventional Gram staining. Gram negative or variable colonies were selected and microbiological tests were performed according to the published surveys (16, 17). The tests are summarized in Table 1.

**Table 1.** Characterization tests for *K. xylinus* identification

**Characterization tests**	***K. xylinus***
Catalase	+
Oxidase	-
Indole production	-
Sodium citrate utilization	-
Methyl red	-
Voges-Proskauer	-
H2S formation	-
Urea utilization	-
Cellulose production	+
Growth on 3% (v/v) ethanol in the	-
presence of acetic acid 5–8%	
Gelatin liquefaction	-
Requirement of acetic acid for growth	-
Growth on malachite-green 0.01% agar	-
Growth on the medium of CaCO3	v
Growth at pH 2	+
Acid formation from glucose	+
Acid formation from sucrose	v
Acid formation from fructose	v
Acid formation from lactose	v
Acid formation from maltose	v

### Cellulose formation and detection.

Seeds of 43 isolated bacteria were inoculated into 100 ml of the HS broth medium and were incubated at 30°C for 7 days. White gelatinous pellicle appeared in the interface of air and the medium was collected. It was treated with NaOH 1% solution at 90°C for 15 min (18, 19). This process was carried out in order to remove other polysaccharides attributed to *Acetobacteraceae*. Only cellulose is the material which is able to resist in that NaOH concentration subjected to boiling. In the present survey, cellulose pellicle was also detected using a fluorescent rapid stain (SIGMA Calcofluor White Stain) which specifically bind to cellulose and is visualized using fluorescent microscope (1, 20). The staining process was carried out according to the manufacture’s instruction.

### Molecular analyses: DNA extraction.

Three methods of boiling, phenol-chloroform and sonication were used as described below for chromosomal DNA extraction of the isolated bacteria. A loopful of the colonies was washed with 1 ml of phosphate buffered saline (PBS, pH 7.6). The pellet was suspended in double distilled water for further procedures.

For boiling procedure the suspension was boiled for 10 min at 85°C. The lysate were centrifuged at 15000 rpm for 5 min. The supernatant containing bacterial DNA was used as template for PCR.

For the second one (phenol-chloroform), 600 μl of extraction buffer (1 M NaCl, 1 M Tris-HCl and 0.05 M EDTA), 13 μl of 25% SDS (sodium dodecyl sulfate) and 3 μl proteinase K (20mg/ml) was added to the washed pellet and incubated for 1 h at 60°C. Equal volume of phenol: chloroform: isoamyl alcohol solution was added to the previous mixture and it was centrifuged at 4°C in 14000 rpm for 10 min. 200 μl of the aqueous phase was separated and 620 μl of chloroform was added. After centrifugation as mentioned above, 2 volumes of cold isopropanol (−20°C) were added to the mixture. The mixture was incubated for 2 h at −20°C. DNA was precipitated with adding 70% ethanol and subsequently dried in air. The quantity of the DNA dissolved in the double distilled water was measured with NanoDrop at 260 nm.

For the third method, sonication process was optimized based on following procedure: the cell suspension was sonicated with 6 pulses at 37 kHz, amplitude of 60 and 30 sec intervals for cooling. Cell debris was removed with centrifugation at 15000 rpm for 5 min.

### DNA amplification.

Based on the genes sequences recorded in databases and alignments performed with softwares such as MEGA 5 and Clustal Omega, five specific primer pairs were designed for amplifying 16S rRNA and cellulose synthase catalytic subunit *(bcsA)*. The primers and the expected region for amplification showed the maximum similarity with the available previous published sequences of *K. xylinus*. The data about oligonucleotide primers are summarized in Table 2. Also the table includes the data about universal primers (27F/1492R) for 16S rRNA and degenerate primers for cellulose synthase which were adopted from previous published surveys (1).

**Table 2.** Primer pairs used for 16S rRNA and cellulose synthase catalytic subunit *(bcsA)* gene detection

**Primer**	**Sequences (′3 → ′5)**	**Product Size (bp)**
16S-1	F: GAGGAACCTGCGTTCGATTAG	436
	R: TACACTGGGAATTCCACAACC	
16S-2	F: GAGGAACCTGCGTTCGATTAG	196
	R: AAAACCTTCTTCACACACGC	
16S-3	F: TGCGTTCGATTAGCTAGTTGG	267
	R: GCTGCTGGCACGAAGTTAG	
Universal	27 F: AGAGTTTGATCMTGGCTCAG	1450
	1492 R: CGGTTACCTTGTTACGACTT	
*bcsA*-1	F: TCCATATCGGGCAGCGCGTG	189
	R: CCCAGGAACAAGAACGCCAGC	
*bcsA*-2	F: GTGCCTCAGCTATTTCCAGA	437
	R: AAATGGTGCGGCGTCTGC	
Degenerate primers	F: CAYGCMAAGGCSGGTAAY	492
	R: CATSCCRCGBGCCCAGCG	

Further molecular analyses were performed with two housekeeping genes: the phenylalanyl-tRNA synthase alpha subunit *(pheS)* and the RNA polymerase alpha subunit *(rpoA)* as described by Naser et al. (2007). Primers of *rpoA* (F: ATGATYGARTTTGAAAAACC / R: ACYTTVATCATNTCWGVYTC) and *pheS* (F: CAYCCNGCHCGYGAYATGC / R: GGRTGRACCATVCCNGCHCC) were used.

PCR was carried out at final concentration of 25 μl. The assay was performed on many occasions under a wide range of conditions for optimizing. Primer concentrations, reaction components, annealing temperature were the factors changed for optimizing and different types of PCR methods like semi-nested and touch-down PCR were used. Most of the assays were generally performed in three-step temperature cycling programs for each primer pairs. The PCR products were run in agarose gel 2% and were visualized with SYBR Green dye. The PCR products’ lengths were estimated with ladder 100 (VIOGENE). To confirm the fragments obtained from the PCR as-says, RFLP-PCR was carried out for the 267 bp and, semi nested PCR and sequencing analysis were performed for the 1450 bp PCR product, respectively.

### RFLP analysis.

In this study, the *TaqI* and The *FokI* restriction enzymes were able to identify and digest the 267 bp-amplified 16S rRNA. The restriction fragments were separated on agarose gel 3.5% and were stained with SYBR Green dye. The length of fragments was compared to the theoretical profiles available in databases.

### Semi-nested PCR.

Two approaches of semi-nested PCR were designed with the combination of designed and adopted primers with regards to the primers location on published sequences. Briefly, the product (1450 bp) obtained using 16S rRNA universal primers was used as a template for the second reactions while it was diluted to 1:10^3^ and 1:10^6^. List of primers order, expected size after amplification and reaction condition are summarized for each approach in Table 3. PCR products were observed after electrophoresis on agarose gel 2% and staining.

**Table 3.** Primers, expected size and reaction condition for semi-nested PCR

**Primer**	**Sequences (′3 → ′5)**	**Product size (bp)**	**PCR condition**
27 F 16S-3 R	AGAGTTTGATCMTGGCTCAG GCTGCTGGCACGAAGTTAG	477	95°C for 2 min (1 cycle); 95°C for 30 sec, 58.1°C for 30 sec, 72°C for 50 sec (30 cycles); 72°C for 5 min (1 cycle)
16S-3 F 1492 R	TGCGTTCGATTAGCTAGTTGG CGGTTACCTTGTTACGACTT	1270	95°C for 2 min (1 cycle); 95°C for 30 sec, 56.5°C for 30 sec, 72°C for 130 sec (30 cycles); 72°C for 5 min (1 cycle)

### Sequence analyses.

PCR products of the 16S rRNA, *pheS* and *rpoA* were purified and sequenced for each strain including the reference strain. These sequences were analyzed using the BLAST program and the GenBank databases.

## RESULTS

Convenient primary screening methods for distinguishing *K. xylinus* from other cellulose producing bacteria were designed using several previous published surveys. The process of *K. xylinus* identification from other cellulose producers was narrowed down using well-organized test series and the strains were confirmed using tests listed in the Table 1. Briefly, collected samples according to their colony formation and cellulose production on HS medium were Gram stained. The negative or Gram variable colonies were isolated for further investigation. The catalase and the oxidase tests were performed. Catalase positive and oxidase negative isolated bacteria were suspected to be one of *Acetobacteraceae* members, therefore isolated strains were prepared for the biochemical and physiological tests.

**Fig. 1.**
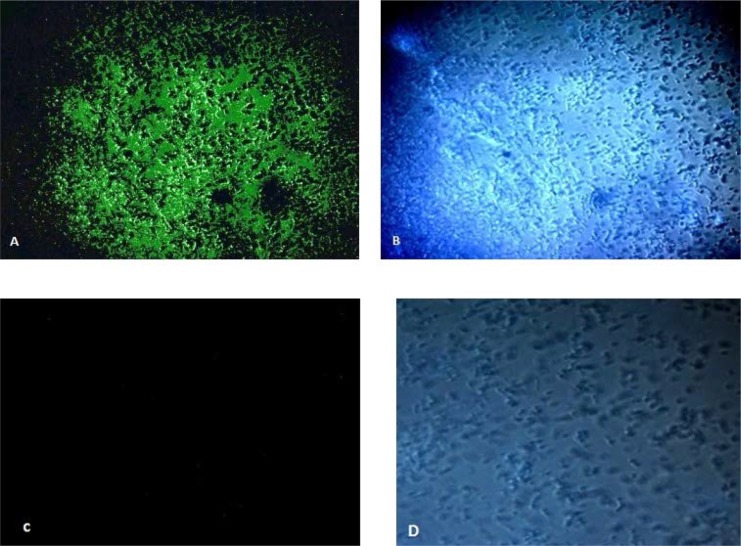
Calcofluor White staining for cellulose detection, 1-*K. xylinus*: A) the dye specifically binds to cellulose and is visualized using UV light, B) The presence of *K. xylinus* was detected by white light, 2-*E.coli* (negative control): C) It is not a cellulose producer bacterium so cellulose were not detected by UV light, D) The presence of the bacteria was visualized using white light

Cellulose was detected with inoculation of the isolated bacteria in the HS broth medium for 7–10 days. A cellulosic biofilm was observed on the interface of air and liquid of the medium. As AAB have been described to produce other exopolysaccharides, the secreted biofilm was boiled in the NaOH 1%. Cellulose is the exopolysaccharide which can resist in the mentioned condition. In contrast, the other exopolysaccharides were removed with this technique. In addition, cellulose was confirmed by Calcofluor white staining. The dye which is specifically binds to cellulose is observed using fluorescence microscope (Fig. 1).

The isolated strains were removed from the trial if they showed variable reaction for individual biochemical and physiological tests or they were not cellulose producer strains. Finally, 43 strains were carefully considered *K. xylinus*.

**Fig. 2.**
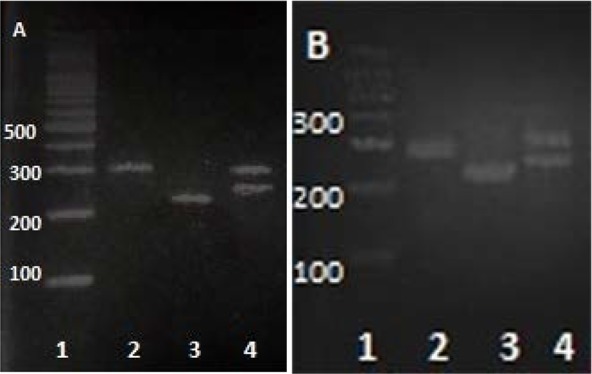
Patterns of PCR products of the 267 bp fragment before and after digestion with the restriction enzymes. A) Reference strain, B) Isolated strain; 1: ladder (100 bp), 2: PCR product before digestion, 3: PCR product after digestion with *FokI*, 4: PCR product after digestion with *TaqI*.

Three different techniques were tested for optimizing the DNA extraction for each isolates. No significant differences were observed in amplification using the techniques. Although many different conditions were examined for each primer pairs, amplification was successfully observed only for 16S-3 primers with expected fragment size of 267 bp. The fragment 267 bp were analyzed with RFLF-PCR using *TaqI* and *FokI* restriction endonuclease. Both enzymes were able to digest the fragment in predicted region based on profiles available in databases. The changes in the fragment size after incubation confirmed the site on which digestion was carried out. Observing RFLP profile comparing to the reference strain confirmed the isolated strains. The patterns of the PCR product before and after digestion with the restriction enzymes are shown in Fig. 2.

**Fig. 3.**
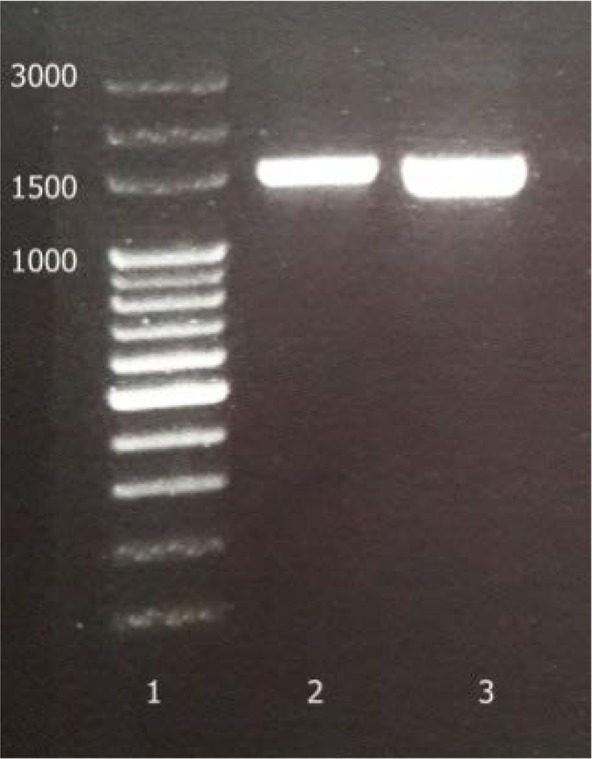
PCR product of the amplified 16S rRNA; 1: ladder, 2: reference strain, 3: isolated strain.

**Fig. 4.**
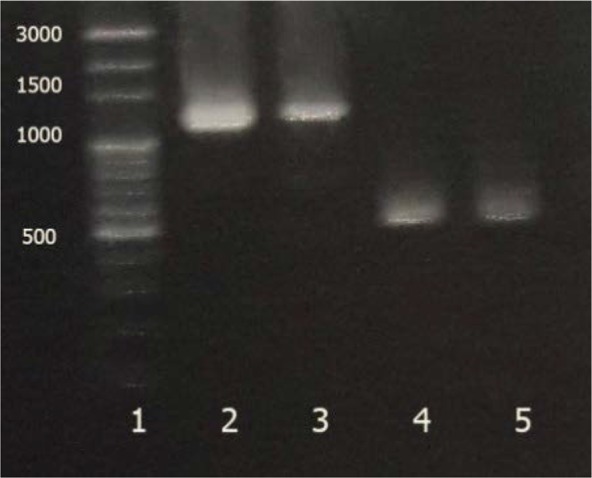
Results of Semi-nested PCR; 1: ladder, 2: fragment 1270 bp for reference strain, 2: fragment 1270 bp for isolated strain, 3: fragment 477 bp for for reference strain, 4: fragment 477 bp for isolated strain.

**Fig. 5.**
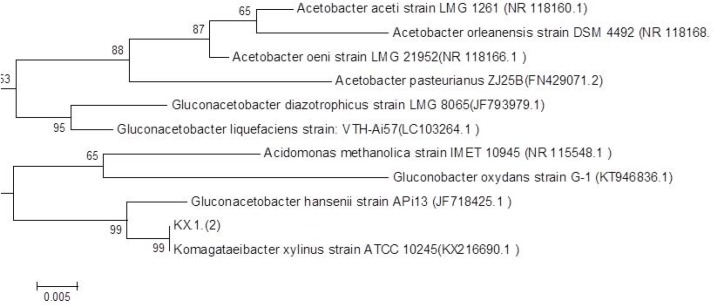
16S rDNA Phylogenetic Tree; isolated strain is shown as KX.1.(2), GenBank accession number for various species is shown in parentheses

Two pairs of primers adopted from the previous published surveys were used for PCR in the selected strains, the universal primers for the 16S rRNA amplification and the degenerate primers for detecting the presence of cellulose synthase gene. The universal primers generated the fragment of 1450 bp (Fig. 3). The semi-nested PCR were designed and the fragments 1270 and 477 were achieved as they had been predicted on the basis of profiles in databases. The results are shown in Fig. 4. For further analysis of the fragment of 1450 bp, it was purified and sequenced (GenBank accession no. KY711526.1). Sequence analysis using BLAST program showed highest similarity (99%) between sequences obtained from current isolated bacteria in this study and registered ones. 16S rRNA phylogenetic tree was constructed using the neighbor-joining method based on 1000 bootstrap. Fig. 5 exhibits the phylogenetic analysis. According to molecular techniques, such as PCR amplification, 16S rRNA sequencing, RFLP-PCR and phylogenetic analysis, the isolates were accurately identified as *K. xylinus.*

**Fig. 6.**
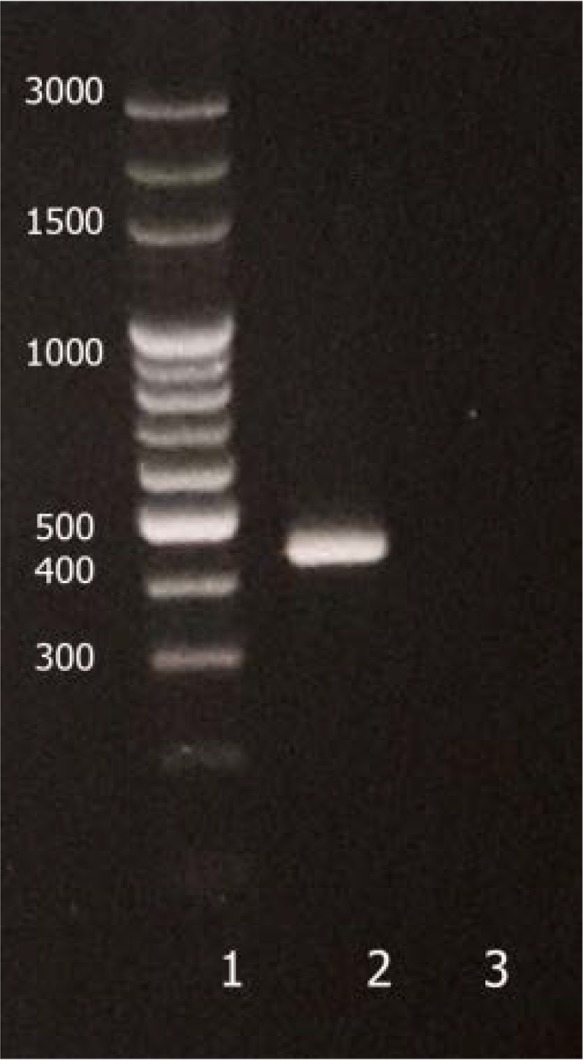
PCR assay for *bcsA* gene; 1: ladder, 2: cellulose-non-producing strain, 3: cellulose producing isolate.

Although many attempts had been made to design homologous primer for the gene *bcsA* and three different protocols in DNA extraction were used, no specific bands were observed. PCR reaction was performed with degenerated primers for this region which is adopted from recent published study. No amplifications were observed for the degenerate primers, while the strains had produced a large amount of cellulose according to the previous described tests. In contrast, one of the cellulose-non-producing strains showed amplification for the desired sequence (Fig. 6).

Fragments of *rpoA* (821 bp) and *pheS* (431 bp) were observed and sequenced. Sequences of *rpoA* gene of our isolated bacteria showed 100% similarity to *rpoA* gene sequences of *Enterococcus faecium* and *pheS* gene sequences showed 99% similarity to *pheS* gene sequences of *Bacillus subtilis* in databases.

## DISCUSSION

According to several published surveys, AAB are commonly associated with fruits, vegetables and beverages like vinegars (2, 4, 22, 23). Beverages and traditional vinegars are considered to be suitable sources of AAB because these vinegars are not filtered (2, 6). Nanda et al. characterized strains responsible for vinegar production from rice vinegar (21). Aydin et al. isolated cellulose producing bacteria from wastes of vinegar in 2009. They did several biochemical and physiological tests to identify the strains (27). Perumpuli et al. reported AAB isolation from coconut water. To their report, identification process was carried out using bacteriological tests (6). In this study, we isolated and identified *K. xylinus* from traditional vinegars using bacteriological and molecular methods.

Techniques such as 16S rRNA analysis and RFLP are considered as appropriate methods for better understanding of classification of microorganisms (24, 25). They are used for genus and species characterization (26–28). For more confirmation of the amplified products, semi-nested and RFLP-PCR was carried out for the 1450 bp and the 267 bp fragments, respectively. To identify the species of the isolated bacteria, 1450 bp PCR products were sequenced to compare with databases. According to their sequences and the results of the BLAST program, there were 99% similarities between the isolated and the registered strains available in databases. That was a high similarity degree which was explained that the isolated strains were *K. xylinus.* According to report of Mounir et al. AAB strains can be isolated from local vinegars of Moroccan in 2016. They sequenced 16S rRNA amplified fragment. To their report, isolated *K. xylinus* revealed 98% homology with *K. xylinus* using BLAST program (5). We achieved the same result on the basis of 16S rRNA and analyzing the phylogenetic tree with related species. We also used RFLP-PCR and semi-nested PCR for more confirmation. RFLP analysis of 16S rRNA amplified gene has been reported as a proper technique for the differentiation and characterization of microorganisms. Ruiz et al. and also Poblet et al. differentiated AAB using the RFLP-PCR of 16S rRNA amplified sequences; however, they could not recognize *Ga. liquifacince, Ga. xylinum* and *Ga. europiance* (26, 29, 30). The same reports are published by previous study (28, 31). In the current study, the isolated strains were identified by comparing RFLP pattern with the reference strain using *TaqI* and *FokI* restriction enzymes for the fragment 267 bp.

Specific genes required for cellulose formation encode via more than one operon named bcs operons. The bcs operons are classified in three major types and several subtypes. Genomic research describes remarkable diversity in subunits of operons, genes sequences and even products. One of the genes which can be found in the majority of mentioned subunits is *bcsA* (32–35). The gene amplification was performed using the designed and the degenerate primers in order to detect cellulose synthase gene in the isolated bacteria. One of the cellulose non-producing strains was positive, however, the cellulose producing strains showed no amplification. This is similar to the result which had been recently reported (1). This may have been due to other genes are active in some strains according to the reported study of Römling and Galperin. Another reason is probably due to the high biological diversity of genes sequences. This is mentioned by previous studies. In the present study, it was revealed that even with observing positive results, it does not approve the presence of cellulose producing bacteria in samples. The genes participated in the cellulose biosynthesis are widespread among the broad range of bacteria but few bacteria can produce cellulose as a secreting material (1, 2, 35).

There was no published data about *pheS* and *rpoA* sequences of *K. xylinus* in databases, however, high similarity between our isolated bacteria, *Enterococcus faecium* and *Bacillus subtilis* can be considered as another proof for biodiversity of *K. xylinus.*

In conclusion, this is the report of *K. xylinus* isolation from Iranian traditional vinegars. Molecular methods were performed according to analysis based on the sequencing of the gene coding for 16S rRNA, *bcsA, rpoA* and *pheS*. According to the results, different molecular techniques expand our knowledge about discriminating bacterial population but appropriate physiological and biochemical methods should be performed for accurate identification especially for those isolated strains from natural samples. Molecular methods should be developed to increase our knowledge especially in the fields of AAB.
